# Editorial: Early life stress and its impact on physiological fitness

**DOI:** 10.3389/fphys.2022.1037409

**Published:** 2022-10-12

**Authors:** Adriana L Burgueño, Mariana Astiz, Alexies Dagnino-Subiabre

**Affiliations:** ^1^ Instituto de Investigaciones Biomédicas, Consejo Nacional de Investigaciones Científicas y Técnicas (CONICET)-Pontificia Universidad Católica Argentina, Buenos Aires, Argentina; ^2^ Center of Brain, Behavior and Metabolism (CBBM), Institute of Neurobiology, University of Lübeck, Lübeck, Germany; ^3^ Laboratory of Stress Neurobiology, Centre for Neurobiology and Integrative Pathophysiology, Institute of Physiology, Faculty of Sciences, Universidad de Valparaíso, Valparaíso, Chile

**Keywords:** stress, development, physiology, prenatal, postnatal

Our physiology is modified by the environment during critical periods of development from gestational to infancy, even in some cases during adolescence. Stressful experiences are often a challenge to physiological homeostasis. Thus, when exposure to stressful experiences occurs during critical periods such as embryonic development or early life, these experiences have a “programming” effect on the health, changing the developmental trajectory and generating long-lasting changes in the structure and/or function of different organs and systems (Zambrano et al., 2016). Early life stress leads to physiological alterations, from metabolism to behavior evidenced by several studies in humans and animals. The effect of maternal starvation from preconception to early childhood, such as in the Dutch famine of World War II, or exposure to stressful events in the Holocaust, has shown associations between early stress and later obesity, and metabolic and behavioral disorders (Painter et al., 2006; Roseboom et al., 2006; Flory et al., 2011). Research on stress has focused on understanding the mechanism of the stress response as well as the specific effects of stress on individuals, using several animal models (Jaggi et al., 2011). Several of these studies have shown important features, one is that the effects of early life stress are also affecting subsequent generations through epigenetic inheritance.

Epidemiological, experimental and literature review studies as those included in this collection are essential to understand the impact of the early environment on the wide spectrum of systems that end up ruling physiological fitness.

Maternal care is clearly one relevant stress factor. In this Research Topic, Dumas reviews the history of maternal care research, highlighting the importance of this key social interaction shaping adult mental and physical health.

Ding et al. conducted a brain imaging study on left-behind children in China. They demonstrated that left-behind children, in addition to deficits in their social-emotional skills, show a decrease in synchronization strength and asymmetry in the right middle frontal gyrus during joint attention. The authors suggested that those might be vulnerability factors in the development of left-behind children. In line with this, Leon el al. provides the first evidence of mother-child epigenetic mark of neglectful caregiving. Nine differentially methylated regions (DMR) were found in common among mothers with neglectful and neglected children, some DMRs contained genes related to childhood adversity, neonatal and infant diabetes, obesity, hypertension, posttraumatic stress, and cancer.

Stress disrupts aspects of serotonergic signaling in the brain and, conversely, serotonergic drugs can modulate the effects of stress. In addition, serotonin plays an important role in the female reproductive system, especially in the development of follicles and embryos. In this Research Topic, Han et al. studied the effect of pregestational exposure to serotonin, and found evidence suggesting that maternal pre-pregnancy serotonin exposure could affect the development of the offspring, partially through reduction in hormone secretion and placental inflammation.

As more is known about the health consequences of early-life stress exposure, interest in finding interventions to prevent or reverse these effects is growing. The use of enriched environments is one of the most studied methods for this purpose. Corredor et al., report that enriched environment has beneficial effects on anxiety-like behaviors in a sex-dependent manner, suggesting that this system is highly plastic during development and have a time window for potential interventions.

Besides the critical developmental windows, chronic stress is able to trigger or impair some psychological conditions, including anxiety disorders and major depression (Pervanidou and Chrousos 2012; Farr et al., 2014). In this Research Topic, Avalos et al. assessed neurobiological mechanisms underlying the influence of chronic restraint stress on the sensitized response to the psychomotor-stimulating effects of cocaine. The authors shed light on the impairment of glutamate signaling on this phenomenon that would be potentially relevant also in the context of development. Henríquez Martínez et al. evaluated the impact of stress exposure on behavior and regeneration ability using zebrafish. Their findings suggest that there is a relationship between chronic stress, regeneration, and behavior in zebrafish. The authors found that the impact of stress was age-dependent. Interestingly, this study suggests that at least some of the mechanisms underlying the effects of the early environment on physiological fitness could be conserved.

This Research Topic introduces papers that, using different animal models and also human studies, help to clarify some of the mechanisms associated to early-life stress exposure. These findings will be important in order to develop effective treatments for stress-related disorders.
